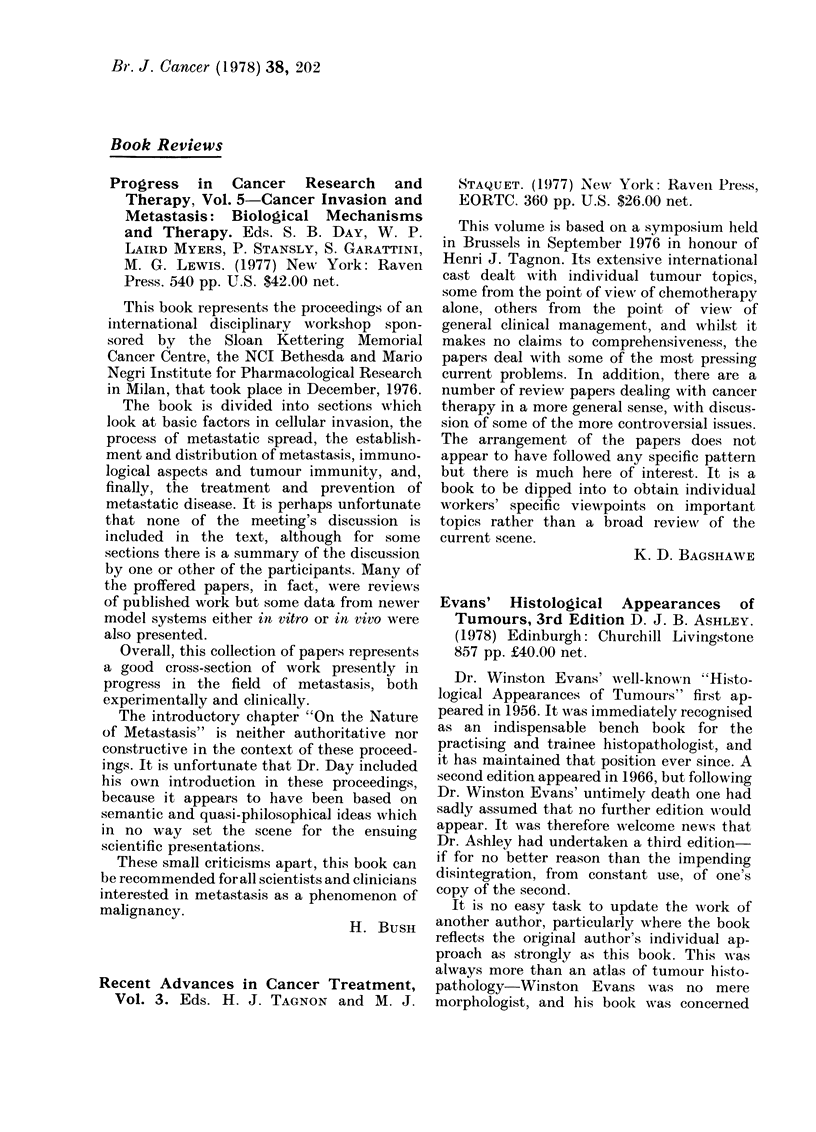# Progress in Cancer Research and Therapy, Vol. 5—Cancer Invasion and Metastasis: Biological Mechanisms and Therapy

**Published:** 1978-07

**Authors:** H. Bush


*Br. J. Cancer* (1978) **38**, 202

### *Book Reviews*

**Progress in Cancer Research and Therapy, Vol. 5—Cancer Invasion and Metastasis: Biological Mechanisms and Therapy.** Eds. S. B. DAY, W. P. LAIRD MYERS, P. STANSLY, S. GARATTINI, M. G. LEWIS. (1977) New York: Raven Press. 540 pp. U.S. $42.00 net.

This book represents the proceedings of an international disciplinary workshop sponsored by the Sloan Kettering Memorial Cancer Centre, the NCI Bethesda and Mario Negri Institute for Pharmacological Research in Milan, that took place in December, 1976.

The book is divided into sections which look at basic factors in cellular invasion, the process of metastatic spread, the establishment and distribution of metastasis, immunological aspects and tumour immunity, and, finally, the treatment and prevention of metastatic disease. It is perhaps unfortunate that none of the meeting's discussion is included in the text, although for some sections there is a summary of the discussion by one or other of the participants. Many of the proffered papers, in fact, were reviews of published work but some data from newer model systems either *in vitro* or *in vivo* were also presented.

Overall, this collection of papers represents a good cross-section of work presently in progress in the field of metastasis, both experimentally and clinically.

The introductory chapter "On the Nature of Metastasis" is neither authoritative nor constructive in the context of these proceedings. It is unfortunate that Dr. Day included his own introduction in these proceedings, because it appears to have been based on semantic and quasi-philosophical ideas which in no way set the scene for the ensuing scientific presentations.

These small criticisms apart, this book can be recommended for all scientists and clinicians interested in metastasis as a phenomenon of malignancy.

H. BUSH